# Axenic green microalgae for the treatment of textile effluent and the production of biofuel: a promising sustainable approach

**DOI:** 10.1007/s11274-023-03863-2

**Published:** 2024-01-29

**Authors:** Ashutosh Pandey, Gaurav Kant, Ashvani Chaudhary, Kaissan T. T. Amesho, Karen Reddy, Faizal Bux

**Affiliations:** 1 Institute for Water and Wastewater Technology, Durban University of Technology, 19 Steve Biko Road, Durban, 4000 South Africa; 2 BiotechnologyBioenergy Research Laboratory, Department of Biotechnology, AKS University Satna, Satna, MP 485001 India; 3 Department of Biotechnology, Motilal Nehru National Institute of Technology Allahabad, Prayagraj, UP 211004 India; 4 Department of Biotechnology, University)IMS Engineering College (Affiliated to Dr. APJ Abdul Kalam Technical University, Lucknow), Lucknow, Ghaziabad, UP 201015 India; 5 Institute of Environmental Engineering, National Sun Yat-Sen University, Kaohsiung, 804 Taiwan; 6 Centre for Emerging Contaminants Research, National Sun Yat-Sen University, Kaohsiung, 804 Taiwan; 7 Centre for Environmental Studies, The International University of Management, Main Campus, Dorado Park Ext 1, Windhoek, 10001 Namibia; 8 Amity Institute of Biotechnology, Amity University Noida Campus, Sec-125, Noida, 201313 UP India

**Keywords:** Wastewater, Phycoremediation, Phylogenetic analysis, *Chlorella sorokiniana* ASK25, Biomass, Biodiesel, Bioethanol, Mass balance

## Abstract

An integrated approach to nutrient recycling utilizing microalgae could provide feasible solutions for both environmental control and energy production. In this study, an axenic microalgae strain, *Chlorella sorokiniana* ASK25 was evaluated for its potential as a biofuel feedstock and textile wastewater (TWW) treatment. The microalgae isolate was grown on TWW supplemented with different proportions of standard BG-11 medium varying from 0 to 100% (v/v). The results showed that TWW supplemented with 20% (v/v) BG11 medium demonstrated promising results in terms of *Chlorella sorokiniana* ASK25 biomass (3.80 g L^−1^), lipid production (1.24 g L^−1^), nutrients (N/P, > 99%) and pollutant removal (chemical oxygen demand (COD), 99.05%). The COD level dropped by 90% after 4 days of cultivation, from 2,593.33 mg L^−1^ to 215 mg L^−1^; however, after day 6, the nitrogen (-NO_3_^−1^) and total phosphorus (TP) levels were reduced by more than 95%. The biomass-, total lipid- and carbohydrate- production, after 6 days of cultivation were 3.80 g L^−1^, 1.24 g L^−1^, and 1.09 g L^−1^, respectively, which were 2.15-, 2.95- and 3.30-fold higher than *Chlorella sorokiniana* ASK25 grown in standard BG-11 medium (control). In addition, as per the theoretical mass balances, 1 tonne biomass of *Chlorella sorokiniana* ASK25 might yield 294.5 kg of biodiesel and 135.7 kg of bioethanol. Palmitic acid, stearic acid, and oleic acid were the dominant fatty acids found in the *Chlorella sorokiniana* ASK25 lipid. This study illustrates the potential use of TWW as a microalgae feedstock with reduced nutrient supplementation (20% of TWW). Thus, it can be considered a promising feedstock for economical biofuel production.

**Graphical abstract:**

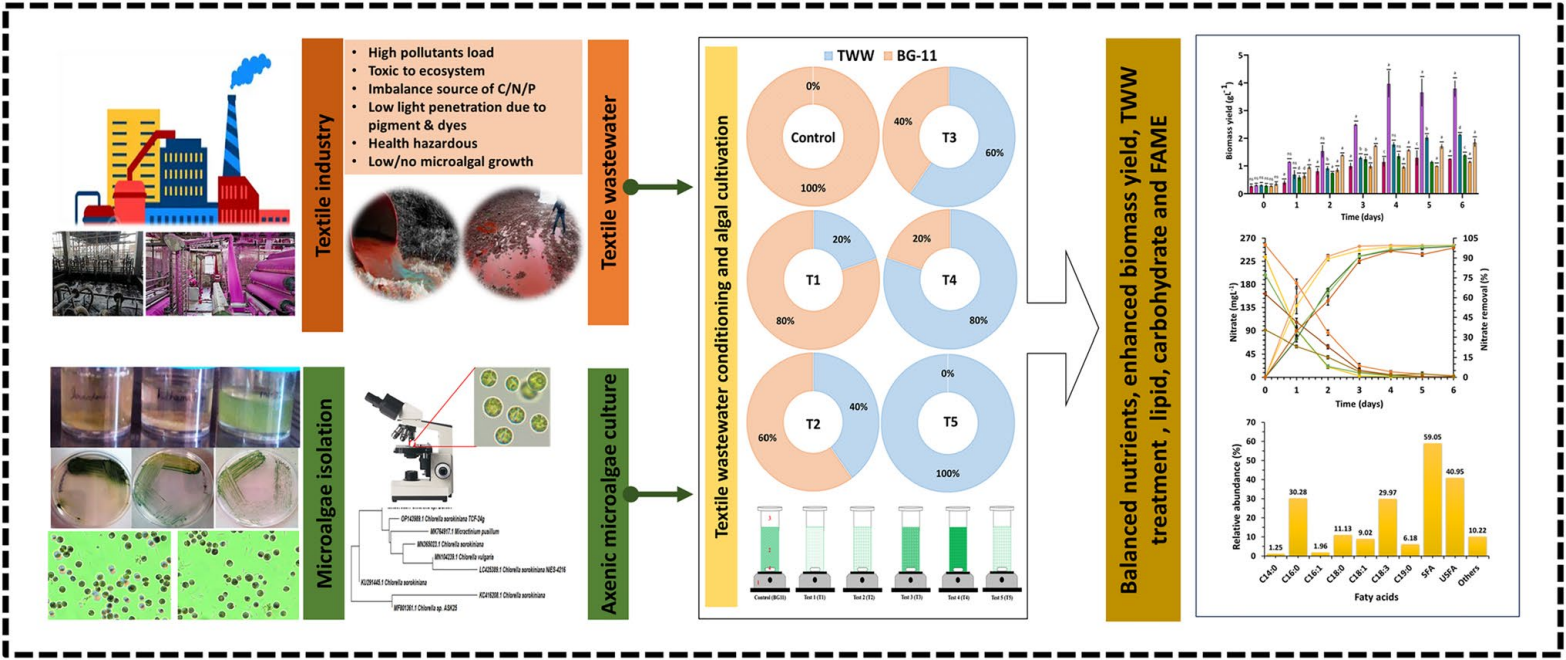

## Introduction

The escalating global population and industrial activity have caused a critical energy crisis. To meet the increasing energy needs, humans have depended on water and fossil fuels such as coal, petroleum, diesel, crude oil, and natural gas (Hussain and Rittmann 2023; Pandey et al. 2019a). Unfortunately, this excessive exploitation of natural resources has raised concerns regarding environmental and ecological sustainability (Thanigaivel et al. 2022). While alternative energy sources exist, they often come with significant drawbacks. Consequently, the production of renewable biofuels has gained substantial attention worldwide. Cultivating microalgae through the phycoremediation method offers a cost-effective and efficient solution to address these challenges (Ugya et al. 2023; Singh et al. 2023a, b). Phycoremediation utilizes macro- or microalgae to reduce or transform pollutants found in wastewater, including harmful substances and nutrients (Vasistha et al. 2023). Microalgae farms serve multiple purposes such as food and fuel production, generation of manure, stabilization agents, reduction of CO_2_ emissions in power plants, and wastewater treatment (Mehariya et al. 2021; Afzal et al. 2023). Moreover, certain species of algae possess the ability to accumulate significant quantities of intracellular lipid, accounting for as much as 60% of their overall biomass (Soth Gao et al. 2023). This lipid accumulation enhances the combustion heat and energy content of algae, further highlighting its potential as a viable energy source.

The utilization of microalgae for wastewater treatment has gained significant momentum due to their effective nutrient removal capabilities and the added advantage of biofuel production. Microalgae exhibit the ability to sequester carbon dioxide, purify wastewater by eliminating pollutants, and generate biofuels, making them a highly promising option (Zhou et al. 2022). Their notable characteristics, such as high biomass production, elevated cellular lipid productivity, and rapid growth rates, further enhance their appeal. Recent studies have also highlighted the potential of utilizing flue gas and combining different wastewater sources for microalgae cultivation (Pandey et al. 2019b). This approach not only improves cost-effectiveness in cultivation but also aids in carbon dioxide sequestration. Choosing an appropriate cultivation method is crucial to improve microalgal output efficiently (Pandey et al. 2019a). Research is ongoing to develop a nutrient-rich and cost-effective microalgal cultivation medium that can enhance wastewater treatment and yield biomass for energy production (Pandey et al. 2020).

The textile industry, among others, consumes a significant amount of freshwater in its processes and generates a substantial volume of wastewater, accounting for approximately 30% of total annual wastewater production (Fazal et al. 2018). Textile wastewater typically contains dyes, heavy metals, nitrates, phosphates, high levels of COD, and sulphates, all of which pose environmental hazards (Yaseen and Scholz 2019). Typically, a dye concentration of 25–2,500 mg L^−1^ is found in TWW. A dye concentration of ≤ 1 mg L^−1^ in wastewater is considered hazardous. These pollutants negatively impact human health, aquatic life, and the overall environment, resulting in eutrophication, carcinogenic effects, and harm to plants and aquatic organisms (Yaseen and Scholz 2019). Therefore, careful consideration of TWW remediation is crucial to achieve higher levels of environmental protection. For the treatment of TWW, physiochemical techniques including coagulation, flocculation, adsorption, and membrane technology are frequently used; however, these techniques are costly, time-consuming, energy-intensive, and produce huge volumes of sludge that must be post-treated before disposal. In recent years, there has been a growing interest in utilizing microalgae for the biological treatment of TWW (Singh et al. 2023a, b; Hashmi et al. 2023). The sustainable production of substantial biomass from untreated TWW not only aids in wastewater disposal but also offers practical opportunities for wastewater treatment and biodiesel production (Fazal et al. 2018; Oyebamiji et al. 2019). However, there are still economic and logistical challenges that need to be addressed before microalgae can be widely employed in industrial wastewater treatment (i) Microalgae's incapacity to flourish in concentrated wastewaters and low biomass productivity are two clear problems. (ii) Concentrated TWW has a high concentration of suspended particulates and dyes, which reduces sunlight transmission and mass transfer and inhibits the growth of microalgae (iii) Energy consumption during the cultivation process and process performance reliability (iv) Optimization of carbon, nitrogen, and phosphorus removal in wastewater, which are crucial nutrients for microalgal growth. To overcome these limitations, the concentrated TWW is diluted with freshwater, nutrients supplementation or pretreatment of TWW to accomplish microalgae growth. It is well established that the nutritional composition of the culture medium significantly influences nutrient assimilation and overall growth rate (Fazal et al. 2018). It is important to establish an appropriate nutrient ratio that aligns with the microalgal elemental stoichiometry to enhance nutrient removal efficiency. This study aims to combine TWW remediation with biofuel production using an axenic microalgal strain isolate, ASK25 (Fazal et al. 2018).

**Fig. 1:**
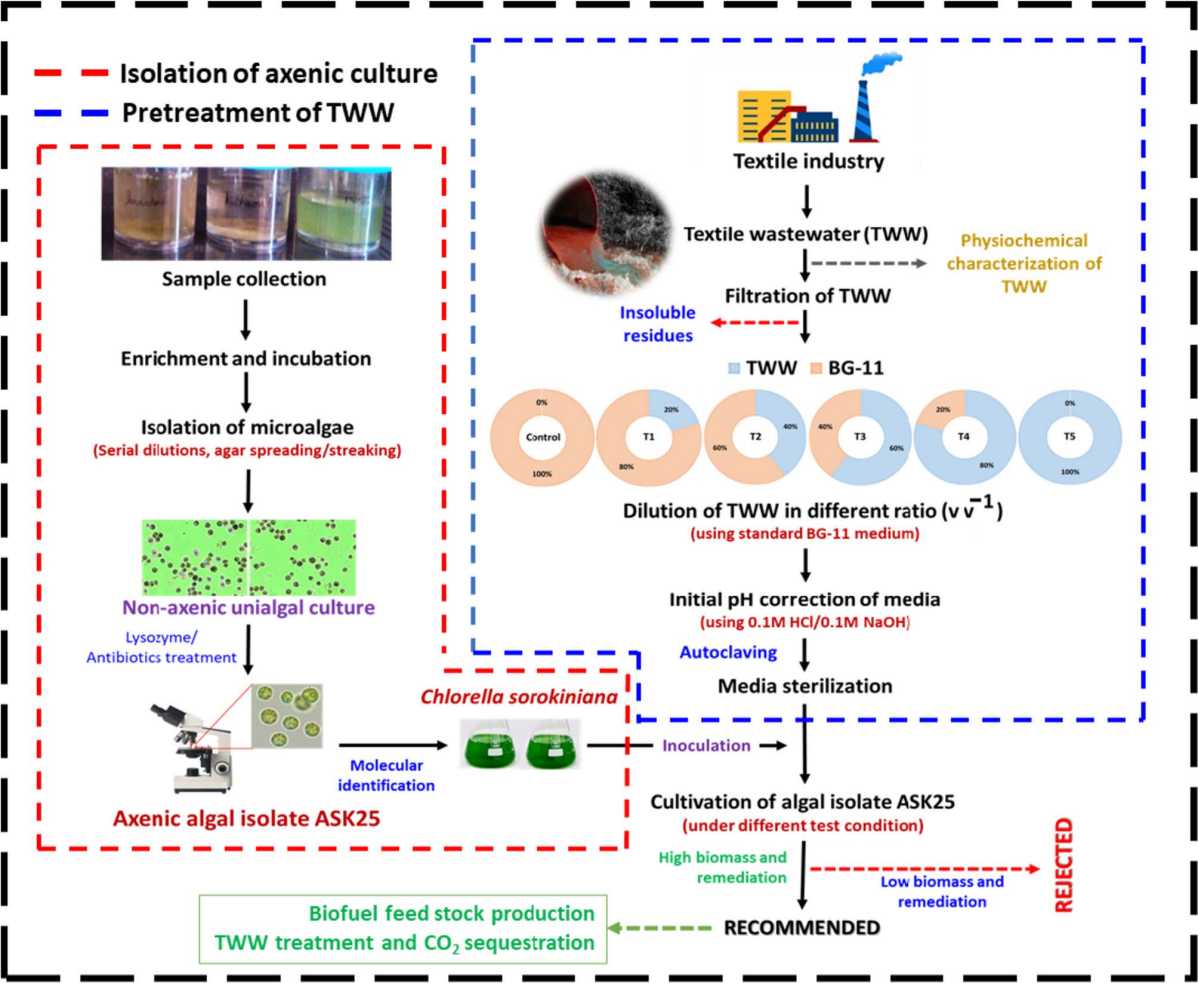
Schematic diagram showing the research plan and pretreatment step of raw TWW (Control: standard BG11 medium, T1 (TWW: BG-11, 1:4); T2 (TWW:BG-11, 2:3); T3 (TWW:BG-11, 3:2); T4 (TWW:BG-11, 4:1) and T5 (TWW:BG-11, 5:0)

The objectives of this study are as follows: (i) to isolate and identify the axenic microalgal strain ASK25 and evaluate its growth under nutrient sequestration from TWW; (ii) to characterize the biomass derived from TWW and assess its potential for biofuel production; and (iii) to analyze the theoretical mass balance for the integrated cultivation of microalgae and remediation of TWW. By addressing these objectives, this study seeks to contribute to the development of a sustainable and cost-effective approach that combines TWW treatment with biofuel production through microalgae cultivation (Fig. 1).

## Materials and methods

### Microalgae strain, maintenance, and its molecular characterization

The axenic microalgae strain used in this study was isolated from a wastewater lodge area situated in Prayagraj, Uttar Pradesh, India. Water samples were collected, enriched and maintained with BG-11 medium: (NaNO_3_ (1.5 g L^−1^), K_2_HPO_4_ (0.04 g L^−1^), MgSO_4_∙7H_2_O (0.075 g L^−1^), CaCl_2_∙2H_2_O (0.036 g L^−1^), citric acid (0.006 g L^−1^), ferric ammonium citrate (0.006 g L^−1^), EDTA (disodium salt) (0.001 g L^−1^), Na_2_CO_3_ (0.02 g L^−1^), and 1 ml of trace element solution from the micronutrient solution containing H_3_BO_3_ (2.86 g L^−1^), MnCl_2_.4H_2_O (1.81 g L^−1^), ZnSO_4_∙7H_2_O (0.22 g L^−1^), Na_2_MoO_4_.2H_2_O (0.39 g L^−1^), CuSO_4_⋅5H_2_O (0.079 g L^−1^), Co(NO_3_)_2_ (0.05 g L^−1^)) (Stanier et al. 1971). The algae were subjected to purification by serial dilutions followed by inoculation into Petri plates containing BG-11 supplemented with 1.5% (w/v) of agar. Single colonies appearing on plates were selected and purified further using lysozyme-antibiotic treatment to get rid of bacterial contaminants from these axenic cultures. In this treatment, algal isolates were placed in Luria Bertani broth supplemented with lysozyme (20 µg mL^−1^) and antibiotic mixture (cefotaxime-500 µg mL^−1^ and tetracycline-50 µg mL^−1^) for 24 h under stationary conditions at 37℃. The cells were then transferred into BG-11 and were incubated for 12 days at 28 ± 2℃ under continuous white light illumination (Sarchizian and Ardelean 2010). The purity of the cultures was ensured by repeated streaking on the nutrient agar plate and routine microscopic examination. Microscopic identification was performed using standard morphological feature keys (Bellinger and Sigee 2015). The initial pH was maintained to 7.2 ± 0.1, and the cultures were grown in Erlenmeyer flasks (2 L) with 1.2 L culture media, under a photon flux of approximately 50–60 μmol m^−2^ s^−1^, photoperiod of 16:8 h at 28 ± 2 °C. Intermittent manual shaking (4–5 times a day) was done to avoid attachment of algal cells on the walls of culture flasks (Pandey et al. 2019b). The complete genomic DNA of the microalgal strain was extracted with HiPurATM Marine Algae DNA purification kit (HiMedia, India) to confirm identification. The genomic region of 18S rDNA was amplified using universal primers (18SF: 5′TCCTGCCAGTAGTCATATGC-3’; 18SR: 5′-TGATCCTCYGCAGG TTCAC-3’) (Pandey et al. 2019a; b). For the polymerase chain reaction (PCR), the reaction mixture consisted of 20 ng of genomic DNA, 1.5 mM MgCl_2_, 0.4 µm primers, 1 unit of Taq DNA polymerase, and 10X buffer (20 mM Tris–HCl, pH 8.4; 50 mM KCl). The PCR protocol consisted of an initial denaturation step at 95 °C for 5 min., followed by 35 cycles of denaturation at 95 °C for 1 min., primer annealing at 50 °C for 1 min., and primer extension at 72 °C for 1 min. A final extension step of 10 min. was carried out to ensure complete amplification. The PCR product was purified using the GeneJET PCR purification kit from Thermo-Fisher and subjected to electrophoretic separation on a 1.0% TAE agarose gel before sequencing. Primers (18SF: 5′TCCTGCCAGTAGTCATATGC-3’; 18SR: 5′-TGATCCTCYGCAGGTTCAC-3′) were used in sequencing reactions, which were examined in an automatic sequencer (ABI PRISM 3730 sequencer, Applied Biosystems). The obtained sequences were then further processed using MEGA 6.0 and Bio-Edit software for phylogenetic analysis and sequence modifications. The nucleotide sequence from the isolated strain of microalgae was uploaded to the Gene Bank (NCBI) and assessed using the BLAST-N tool (NCBI, BLAST, USA) (Pandey et al. 2019a). The phylogenetic tree was constructed using an alignment and 1000 iterations of the bootstrap analysis.

### Characterization of textile wastewater

The TWW was collected from the handloom city of Pilkhuwa-Hapur, located in Uttar Pradesh, India (N 28°42′41.5314″, E 77°39′7.16328″). These facilities, which are small-scale enterprises engaged mostly in printing and dyeing, release their effluent to the land and water bodies nearby without any kind of treatment. To ensure sample preservation, the untreated raw TWW was stored in sterile sampling bottles at a 4 °C. The physical and chemical properties of the TWW were assessed in accordance with the established guidelines provided by American Public Health Association (APHA, 1989). Each parameter was evaluated in triplicate (Pandey et al. 2020).

### Experimental setup and microalgal growth in TWW

**Fig. 2:**
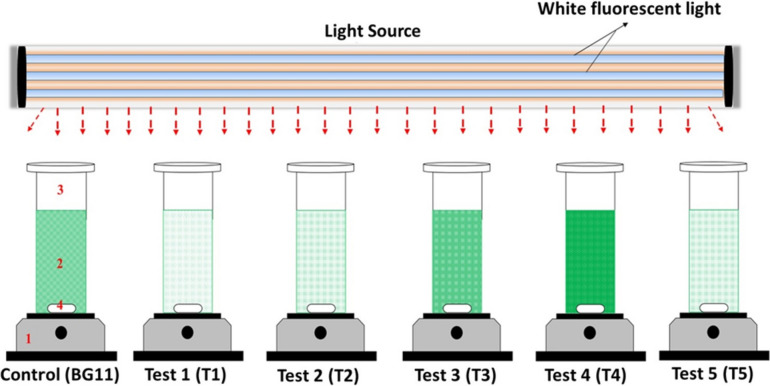
Experiment set to grow algal isolate in different test media. (Control: standard BG11 medium, T1 (TWW: dH_2_O, 1:4); T2 (TWW: dH_2_O, 2:3); T3 (TWW: dH_2_O, 3:2); T4 (TWW: dH_2_O, 4:1) and T5 (TWW: dH_2_O, 5:0). Keys: 1: magnetic stirrer; 2: culture medium; 3: bubble column and 4: magnetic bar

Comparative growth profile of microalgal isolate ASK25 was studied employing varying concentrations of TWW (20, 40, 60, 80, and 100%) diluted with BG-11. The BG-11 growth medium was used as the control. Autoclaved TWW at various concentrations (20, 40, 60, 80 and 100%) was employed to study the growth of the microalgal isolate ASK25 and the initial pH for all the concentrations was adjusted to 6.8 ± 0.2 using 0.1 M NaOH and 0.1 M HCl. Homogenous algal suspension (10%, v/v) was used for inoculation having an optical density of 2.0 at 680 nm. The 4 L bubble columns reactors were used for microalgal isolate ASK25 containing 3 L of working volume employing different concentrations of TWW and BG-11(as depicted in Fig. 2) under a photon flux of approximately 50–60 μmol m^−2^ s^−1^, photoperiod of 16:8 h at 28 ± 2 °C. The growth of the microalgal isolate ASK25 was monitored during 24 h intervals by measuring optical density at 680 nm using UV–visible spectrophotometer (Agilent, Carry 60).

### Evaluation of microalgae growth

Microalgae samples (5 mL) were filtered using GF/C glass fibre filter paper. To determine the dry biomass yield and productivity, the filter sheets containing algal biomass were rinsed twice with ultrapure water and then incubated at 70 °C until a constant weight was achieved. The calculation of biomass produced (g L^−1^) and biomass productivity (g L^−1^ d^−1^) was calculated as the dry biomass produced at late log phase using the following formula (Abomohra et al. 2013).

1 $$ {\text{Biomass}}{\mkern 1mu} {\text{production}}\left( {{\text{gL}}^{{ - 1}} } \right) = \frac{X}{V} $$

2 $$ {\text{Biomass}}\,{\text{productivity}}\,\left( {Bp,{\text{ g L}}^{{ - {\text{1}}}} {\text{d}}^{{ - {\text{1}}}} } \right)\, = \,\frac{{X_{s}  - X_{i} }}{{\Delta t}} $$

where *Bp* was the biomass productivity*, V* was the sample volume, *X* was the weight of the dried biomass, and *Δt* was the batch length (number of days).

### Total Lipid content analysis

The analysis of lipid content was performed using the modified Bligh and Dyer (1959) method for total lipid extraction. In brief, 100 mg freeze-dried microalgae biomass was mixed with a methanol and chloroform solution (2:1, v/v). The mixture was vortexed for 10–15 min. to ensure homogenization and then centrifuged at 7000 × *g* for 10 min. This extraction process was repeated four to six times until the biomass became colourless. The organic solvent containing lipids was filtered through Whatman No. 42 filter paper to remove any remaining biomass and subsequently oven-dried at 55 °C. The lipid content (% wt.) was determined by comparing the extracted lipid weight to the known weight of the dry biomass (Abomohra et al. 2013). The total lipid content and volumetric lipid productivity (mg L^−1^ d^−1^) were calculated by using following equations (Eqs (iii)-(iv)) (Fawzy et al. 2017).

3 $$ {\text{Total}}{\mkern 1mu} {\text{lipid}}{\mkern 1mu} {\mkern 1mu} {\text{content}}\left( {\% ,{\text{wt}}.} \right){\mkern 1mu}  = {\mkern 1mu} \frac{{W_{l} }}{{W_{b} }}{\mkern 1mu}  \times {\mkern 1mu} 100 $$

4 $$ {\text{Lipid}}{\mkern 1mu} {\text{productivity}}{\mkern 1mu} \left( {{\text{mgL}}^{{ - 1}} {\text{d}}^{{ - 1}} } \right) = {\text{Biomass}}{\mkern 1mu} {\text{productivity}}\left( {{\text{mgL}}^{{ - 1}} {\text{d}}^{{ - 1}} } \right){\mkern 1mu}  \times {\mkern 1mu} \frac{{{\text{Lipid}}{\mkern 1mu} {\text{content}}{\mkern 1mu} \left( {\% {\text{wt}}.} \right)}}{{100}} $$

where, *W*_*l*_ and *W*_*b*_ are weight of dry lipid (mg) and weight of dry microalgal biomass (mg), respectively.

### Fatty acid methyl ester analysis

The lipid extracted from the isolated algae species underwent simultaneous esterification and transesterification. Methanol acted as the acyl acceptor while H_2_SO_4_ served as the catalyst. The reaction took place in a water bath at 70 °C for 3 h with a methanol-to-oil ratio of 6:1 (Breuer et al. 2013). This process converted the fatty acids into their corresponding fatty acid methyl esters (Pandey et al. 2022). Hexane and distilled water were used to remove any remaining methanol and acid catalyst from the reaction mixture. The upper organic layer's biodiesel was removed for further examination. The composition of fatty acid methyl esters in *Chlorella sorokiniana* ASK25 was investigated using a gas chromatography (Agilent-7890) (Pandey et al. 2020). The fatty acid composition was analysed following the guidelines by Ostermann et al. (2014), and a NIST library search was conducted to identify the specific fatty acid components.

### Sampling and pollutants removal analysis

A daily sample of 10 mL was collected and centrifuged for 10 min. at 3500 × *g*, then filtered through a 0.45 μm nylon membrane filter. The residual nitrate (-NO_3_^−1^), total phosphorus (TP), and COD were calculated according to Pandey et al. 2020. The percentage removal was calculated using the following equation (Eq. 5).

5 $$ {\text{Nutrients}}\,{\text{removal}}\,\left( \% \right)\, =\,\frac{{C_{i} - C_{s} }}{{C_{i} }}\,\times\,100 $$

where C_i_ and C_s_ were defined as the nutrient concentration at the initial and sampling time, respectively.

### Dye wastewater decolonization study

In the decolourization study, the maximum absorbance of the dye wastewater was assessed. To evaluate the sensitivity of the instrument and typical colour intensities, a diluted solution was scanned across a range of wavelengths. For measuring the maximum absorbance wavelength (λ_max_) of the wastewater sample within the range of 200 to 700 nm, a UV–Vis spectrophotometer was employed. The difference in absorbance measurements before and after the experiment was used to quantify the amount of dye removed. Thereafter, microalgae cells were separated by centrifugation at 3500 × *g* for five min. The absorption spectra of the supernatant were analysed until the absorbance peaks at λ_max_ disappeared (Oyebamiji et al. 2019). The percentage of dye removal was calculated using the following equation (Javed et al. 2022).

6 $$ \% {\text{dye}}\, {\text{removal}}\, =\, \frac{{Absi - Abs_{{f~~}} }}{{Abs_{i} }} \times 100 $$

where, *Abs*_*i*_ and *Abs*_*f*_ initial and final absorbance of water sample, respectively.

### Statistical analysis

The statistical analysis was performed in GraphPad Prism and Microsoft office excel. The samples chosen for analysis were derived from three independent experiments. Data are presented as graphs or in-text showing the mean values ± SD as appropriate.

## Results and discussions

### Identification of microalgae and physicochemical characteristics of TWW

**Fig. 3:**
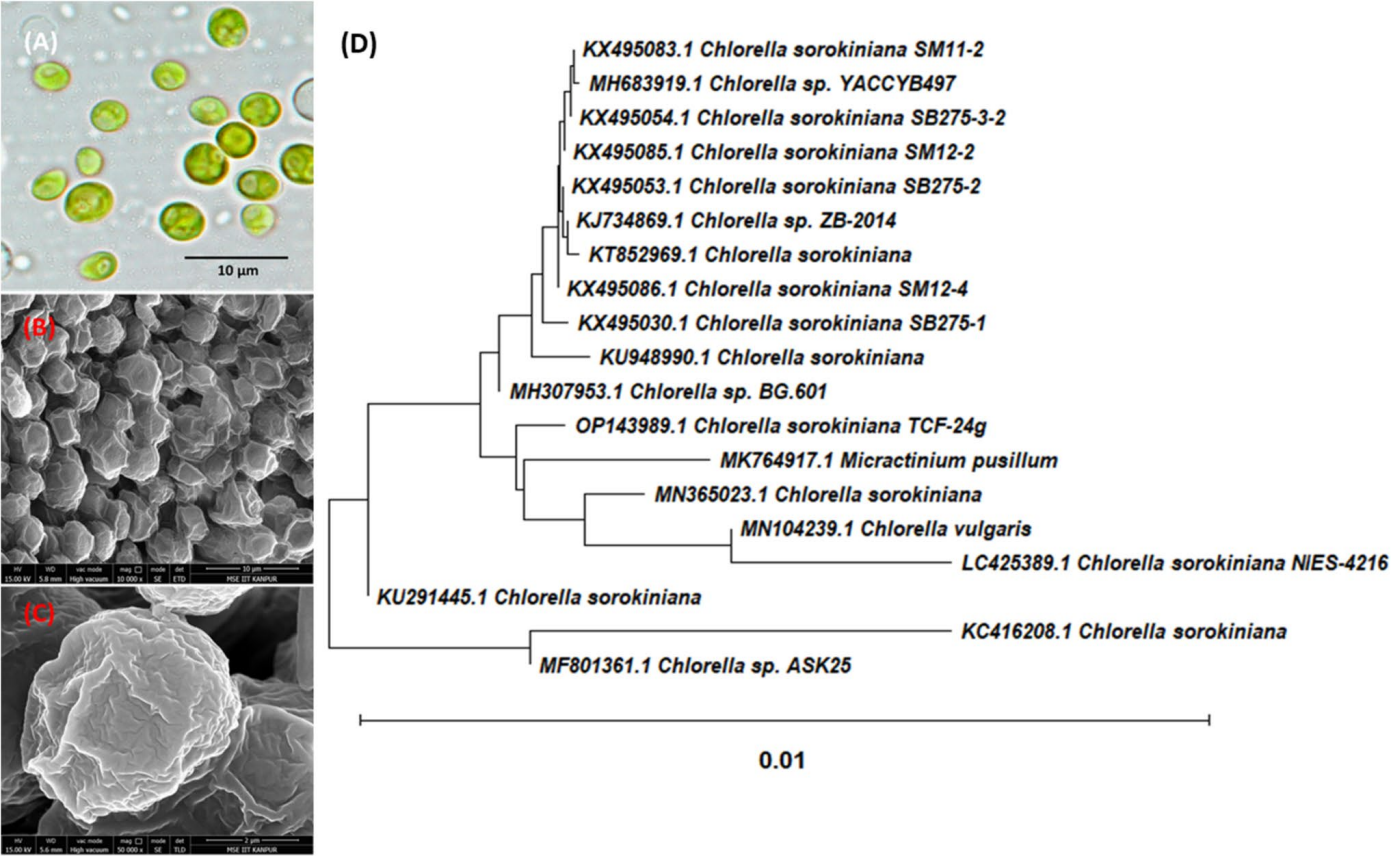
**A**–**C** Phase contrast- and scanning electron microscopy of ASK25 isolate evolutionary relationships of taxa, the evolutionary history was inferred using the Neighbour-Joining method.** D** The percentage of replicate trees in which the associated taxa clustered together in the bootstrap test (1000 replicates)

Through microscopic examination, axenic algal cultures were preliminary classified, according to the colonial nature of the algal isolates (Doppler et al. 2021). The microscopic analysis identified the isolates as belonging to the genus *Chlorella*. *Chlorella* is a type of single-celled green algae ranked under the *Trebouxiophyceae* class and *Chlorellaceae* family (Hodač et al. 2016; Lortou et al. 2022). The cells are solitary, ranging in diameter from 2–10 µm, and have a globular, ellipsoidal, or spherical shape (Fig. 3A–C). They lack flagella and possess a cup-shaped chloroplast with a single pyrenoid, surrounded by a thin cellulose wall (Lortou et al. 2022).

To confirm the accuracy of the morphological observations, phylogenetic analysis was carried out using the partial 18S rRNA gene sequences of the isolated samples (Pandey et al. 2019a). The partial 18S rRNA regions of the isolates were subjected to PCR amplification, and a 900 bp amplicon of the 18S rRNA gene was sequenced. BLAST analysis of the algal isolate's partial 18S rRNA gene sequences revealed a 99% sequence identity with *Chlorella sorokiniana* (Ghaffari and Kalantari 2012). The physical characteristics of the isolate, along with the partial 18S rRNA sequence, confirmed its identification as *Chlorella sorokiniana* ASK25 (Fig. 3D).

The physicochemical properties of the TWW employed in this investigation are shown in Table 1. The pH, total suspended solids (TSS), and total dissolved solids (TDS), of TWW, were 7.91 mg L^−1^, 7,479 mg L^−1^, and 4,252 mg L^−1^, respectively. The COD and biochemical oxygen demand (BOD) were 3,209 mgO_2_ L^−1^ and 1,214 mgO_2_ L^−1^, respectively. Nitrogen and phosphorus are the key nutrients needed for the growth of microalgae. The recorded results for nitrate (N-NO_3_^−1^), ammonia (N-NH_4_^+1^), and phosphate (P-PO_4_^−3^) were 308.5 mg L^−1^, 43.71 mg L^−1^, and 84.43 mg L^−1^, respectively. The sulphates (71.46 mg L^−1^) and other ingredients such as iron (Fe, 0.729 mg L^−1^), copper (Cu, 0.228 mg L^−1^), and zinc (Zn, 4.23 mg L^−1^) observed were recommended to support the growth of algae. The wastewater also contained the presence of heavy metals: lead (0.135 mg L^−1^), cadmium (0.273 mg L^−1^), manganese (1.381 mg L^−1^), nickel (0.627 mg L^−1^).

**Table 1:** Physicochemical characteristics of raw textile industry wastewater

Characteristics	Value
Initial pH	7.90 ± 1.30
Total dissolve solids (TDS)	7479 ± 267*
Total suspended solids (TSS)	452 ± 300*
Nitrate (N-NO_3_^−1^)	308.5 ± 41.50*
Ammonia (N-NH_4_^+1^)	43.71 ± 11.13*
Total phosphorus (P-PO_4_^−3^)	84.43 ± 27.58*
Sulphate (SO_4_^−2^)	71.46 ± 12.56*
Chemical oxygen demand (COD)	3209 ± 17.00*
Biochemical oxygen demand (BOD)	1214 ± 25.16*
Metals	
Cd	0.27 ± 0.03*
Zn	4.24 ± 0.38*
Mn	1.38 ± 0.06*
Ni	0.63 ± 0.02*
Cu	0.23 ± 0.10*
Fe	0.73 ± 0.24*

^*^ mg L^−1^

### Growth evaluation and lipid content of *Chlorella sorokiniana* ASK25 *on different media*

**Fig. 4:**
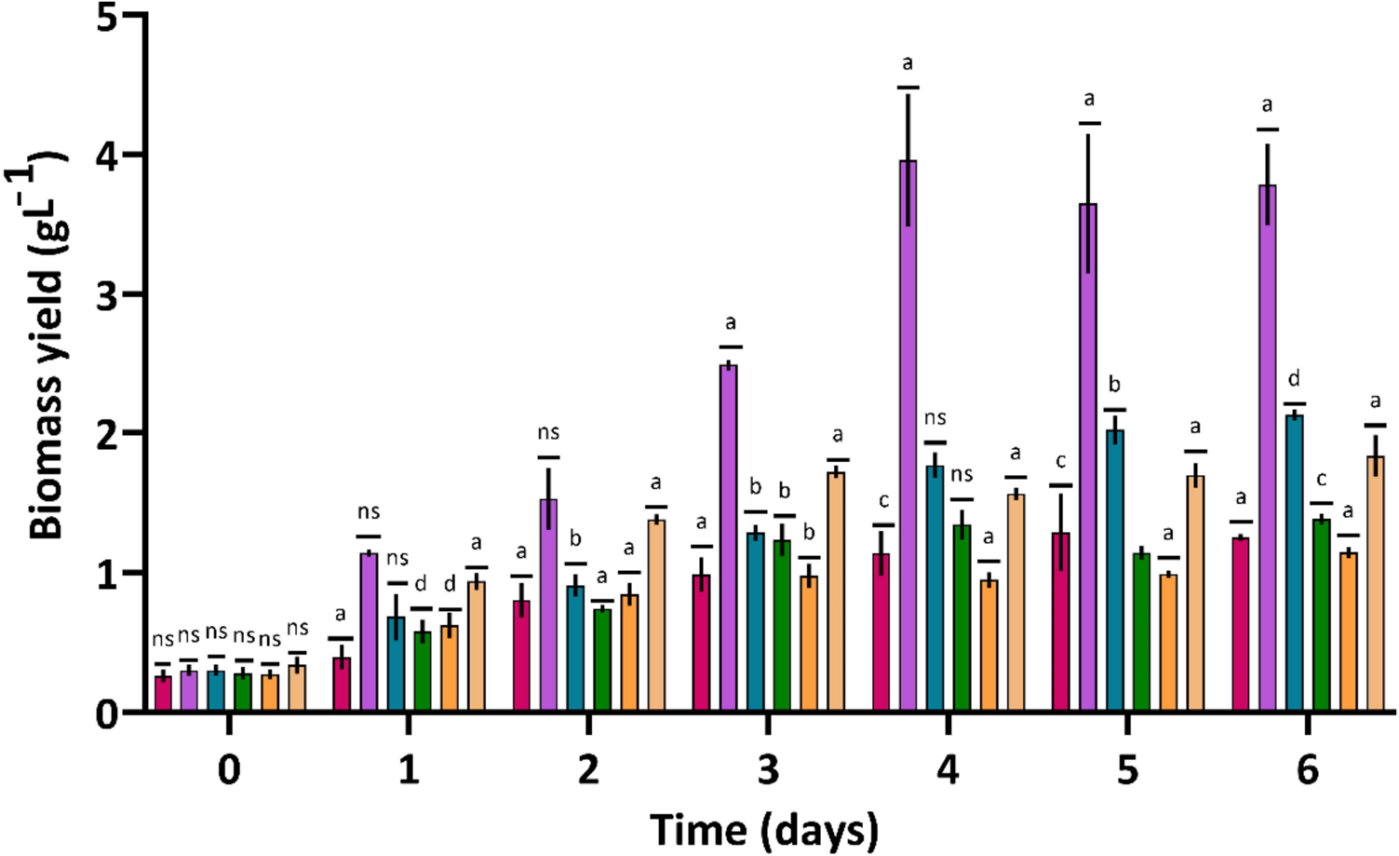
Time course growth profile of *Chlorella* sp. ASK25 under different test conditions (BG11 medium control, T1 (TWW: dH_2_O, 1:4); T2 (TWW: dH_2_O, 2:3); T3 (TWW: dH_2_O, 3:2); T4 (TWW: dH_2_O, 4:1) and T5 (TWW: dH_2_O, 5:0). The graph indicates significant differences (indicated with a-d) in mean values (n = 3) with corresponding adjusted p-values (P < 0.05) providing insights into the impact of different experimental conditions on the response variable (Dunnett's test)

*Chlorella* sp. is frequently employed in microalgae biorefineries for wastewater remediation and the production of valuable bio-products, such as biofuel. The TWW used in this study contained a high concentration of COD (3,209 mgO_2_ L^−1^) and dye (not measured quantitatively). Hence, it was essential to determine whether *Chlorella sorokiniana* ASK25 could thrive and undergo complete decolorization in undiluted 100% TWW (T5), or if it necessitated multiple dilutions with standard-BG-11 medium. *Chlorella sorokiniana* ASK25 was grown in undiluted 100% TWW (T5) and diluted TWW of varying concentrations, including 80% (T4), 60% (T3), 40% (T2), and 20% (T1) shown in Fig. 4. The biomass production of *Chlorella sorokiniana* ASK25 grown in the TWW supplemented with 20% (v/v) standard-BG11 was (T4 medium) 3.80 g L^−1^ in the late log phase, followed by T3 (2.10 g L^−1^), control (1.77 g L^−1^), T2 (1.38 g L^−1^), T5 (1.25 g L^−1^), and T1 (1.15 g L^−1^). The *Chlorella sorokiniana* ASK25 biomass production in the T4 medium was 2.15-, 3.30-, and 3.04-fold higher than control (standard-BG11) T1, and T5 media. On the first day of the cell growth, the growth of *Chlorella sorokiniana* ASK25 showed relatively consistent results across all test condition. However, on the second day, significant differences were observed, possibly due to the dilution of the raw TWW, resulting in different nutrients- and dye concentrations. Moreover, in all treatments, the cells entered into an exponential phase, after 4 days of cultivation, thereafter, the growth slowed down and reached a stationary phase within two days (6 days). Among the test conditions, T4 demonstrated high biomass production, lipid productivity, and nutrient removal (Table 2). In terms of biomass productivity, T4 (0.63 mg L^−1^ d^−1^) showed higher (p < 0.05) productivity than all other treatments (T1, T2, T3, and T5) and control (standard-BG-11). The delayed growth of cells in the T5 condition could be attributed to the high concentration of dye in the medium, which impeded the light's ability to penetrate the medium and the microalgae's photosynthetic activity. In an interesting study by Lim et al. (2010), *Chlorella vulgaris* produced 106.67 mg L^−1^ of biomass when grown in raw TWW (Lim et al. 2010). Similarly, Wu et al. (2017) reported that *Chlorella sp.* produced 137 mg L^−1^ of biomass and 8.6 mg L^−1^ of lipid cultivated in TWW per day (Wu et al. 2017). It was also observed by Bhattacharya et al. (2017) that *Chlorella variabilis* could grow in TWW and get a biomass yield of 74.96 mg L^−1^ with a lipid content of 20.1% (wt.) (Bhattacharya et al. 2017). The biomass generated through phytoremediation of TWW holds potential for various applications, including its use as an energy feedstock for biofuel production (biodiesel or biogas through anaerobic digestion). Moreover, dried biomass can be used as a fertiliser (Pandey et al. 2020; Uddin et al. 2021). These findings indicate that the upscaling of phytoremediation and biomass production holds great potential.

**Table 2:** Growth of *Chlorella* sp. ASK25 in BG-11 medium (control) and different proportions of TWW

Test condition	Biomass yield (g L^−1^)	Biomass productivity (g L^−1^ d^−1^)	Lipid content (%, wt.)	Lipid productivity (mg L^−1^ d^−1^)	Carbohydrate content (%, wt.)	Nutrient removal efficiency (%)			Decolourization efficiency (%)
						COD	TP	Nitrate	
Control (BG11)	1.77^a^	0.30^a^	23.68^a^	69.93^a^	18.71^a^	–	–	–	–
T1	1.15^b^	0.19^b^	28.57^b^	54.57^b^	35.05^b^	96.58^a^	97.70^a^	97.66^a^	98.16^a^
T2	1.38^c^	0.23^c^	26.56^c^	61.04^c^	33.27^c^	98.04^b^	97.79^a^	99.32^b^	97.27^b^
T3	2.10^d^	0.35^d^	23.48^d^	82.06^d^	31.63^d^	98.24^b^	97.93^a^	99.55^b^	98.12^a^
T4	3.80^e^	0.63^e^	32.72^e^	206.95^e^	28.74^e^	99.05^c^	99.94^b^	99.69^b^	99.71^c^
T5	1.25^f^	0.21^f^	21.93^f^	43.57^f^	25.69^f^	94.16^d^	98.61^b^	98.85^b^	96.18^d^

*The results shown here are the average value of three independent experimental observations (standard deviation ≤ 8%). T1-TWW: BG-11, (1:4, v/v); T2-TWW:BG-11, (2:3, v/v); T3-TWW:BG-11, (3:2, v/v); T4-TWW:BG-11, (4:1, v/v) and T5-TWW:BG-11, (5:0, v/v). Mean followed by the same letter in each column are not significantly different p < 0.05 (Tukey tests)

### Pollutant stripping from the different concentrations of TWW by ASK25

The TWW is characterized by its elevated levels of essential nutrients, specifically carbon (C), phosphorus (P), and nitrogen (N). Algae play a crucial role in removing these nutrients from the cultivation environment through nitrogen assimilation and phosphorylation mechanisms (Choi and Lee 2015; Fazal et al. 2021). Nitrogen assimilation involves the conversion of inorganic forms of nitrate, nitrite, and ammonia into their organic forms. They serve as fundamental components for the synthesis of peptides, proteins, enzymes, chlorophylls, energy-transfer molecules such as adenosine diphosphate (ADP) and adenosine triphosphate (ATP), as well as genetic material (RNA and DNA) (Pandey et al. 2019b). The reduction of nitrate and nitrite to ammonium during assimilation is facilitated by nitrate and nitrite reductase enzymes, respectively (Masclaux-Daubresse et al. 2010; Sanz-Luque et al. 2015; Pandey et al. 2019b).

Glutamine (GLN) incorporates nitrogen from ammonium with the assistance of glutamate (Glu) and ATP. These compounds play a vital role in enabling the integration of ammonium into intracellular GLN. Simultaneously, through the process of phosphorylation, inorganic phosphorus (H_2_PO_4_^−^ and HPO_4_^−2^) is added to intracellular organic substances like proteins, lipids, and nucleic acids (Newsholme et al. 2003; Sanz-Luque et al. 2015; Fazal et al. 2021). Microalgae possess several phosphate transporters on their plasma membrane to uptake inorganic phosphorus for cellular transformation (phosphorylation). This transformation involves ATP production from ADP and the synthesis of polyphosphate-by-polyphosphate kinase, occurring in the presence of light during photosynthesis. Dye bath additives, printing (-NH_4_^+1^), coating preparation, dyeing, and textile wet processing are the primary sources of nitrogen and phosphorus in TWW (Masclaux-Daubresse et al. 2010; Sanz-Luque et al. 2015).

**Fig. 5:**
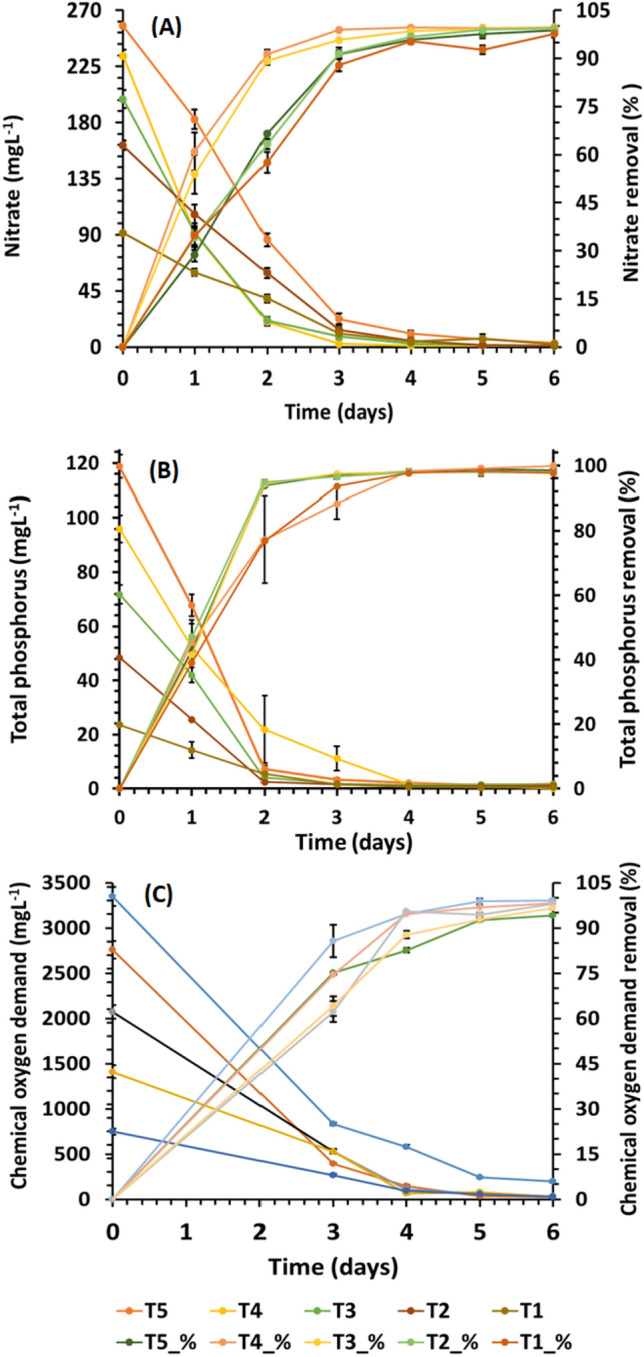
Nutrients and COD removal profile under different test conditions

The green microalgae *Chlorella sorokiniana* ASK25 utilized the nutrients present in the TWW for its growth (Fig. 4). The nutrient removal profile is shown in Fig. 5. The nitrate (-NO_3_^−1^) removal efficiency at the end of day 4 of the batch experiment was 95.81% (from 257.34 mg L^−1^ to 10.79 mg L^−1^), 99.54% (from 233.73 mg L^−1^ to 1.07 mg L^−1^), 98.40% (from 203.98 mg L^−1^ to 3.45 mg L^−1^), 95.68% (from 162.84 mg L^−1^ to 7.04 mg L^−1^) and 94.47% (from 92.42 mg L^−1^ to 5.11 mg L^−1^), the orthophosphate removal efficiency was 98.17% (from 115.7 mg L^−1^ to 2.44 mg L^−1^), 97.56% (from 92.42 mg L^−1^ to 7.51 mg L^−1^), 97.45% (from 69.42 mg L^−1^ -1.47 mg L^−1^), 97% (from 46.28 mg L^−1^ to 0.98 mg L^−1^) and 92.34% (from 23.14 mg L^−1^ to 0.49 mg L^−1^) for T5, T4, T3, T2, and T1, respectively. *Chlorella sorokiniana* isolates, such as ASK25, efficiently utilize available resources to undergo various physiological processes, resulting in the production of biomass rich in protein, carbohydrates, and lipids. When cultivated in open ponds under natural conditions, *Chlorella vulgaris* cultured in TWW showed a reduction in nitrogen and phosphate levels by approximately 45% and 33% respectively. However, variations in pH, temperature, and light intensity during the cultivation process may have affected the efficiency of nutrient removal (Lim et al. 2010). On the other hand, *Chlorella sp.* demonstrated a high nitrogen removal efficiency of 78% in TWW, indicating that the effectiveness of nutrient removal is influenced by factors such as inoculum size, microalgae physiology, and cultivation conditions (Wu et al. 2017). It is important to maintain an optimal N/P ratio in the wastewater to achieve the highest level of efficiency in nitrogen and phosphorus removal (Fazal et al. 2021).

COD is an important parameter for assessing the quality of TWW, as with any other type of wastewater (Munck et al. 2015). The various textile processing steps such as desizing, scouring, bleaching, dyeing, and printing contribute to elevated COD levels, which can lead to a decrease in dissolved oxygen in the water (Işık and Sponza 2008; Azimi et al. 2021). Microalgae play a crucial role in reducing the load of contaminants by sequestering both organic and inorganic pollutants. Certain *Chlorella* species can convert azo dyes into less complex compounds or CO_2_ through metabolic processes, effectively eliminating these dyes from the wastewater (Ishchi and Sibi 2020). Azo dyes can provide algae with both carbon and nitrogen, which contributes to the high COD in TWW (Ledakowicz and Paździor 2021). The COD removal efficiency was 82% (from 3210 mgO_2_ L^−1^ to 577.7 mgO_2_ L^−1^), 94.3% (from 2696 mgO_2_ L^−1^ to 153.7 mgO_2_ L^−1^), 94.6% (from 2044 mgO_2_ L^−1^ to 110.4 mgO_2_ L^−1^), 95.44% (from 1420.22 mgO_2_ L^−1^ to 64.76 mgO_2_ L^−1^) and 87% (751.02 mgO_2_ L^−1^ to 97.63 mgO_2_ L^−1^) for T5, T4, T3, T2, and T1 respectively (Fig. 5A–C). In a similar study, it was found that *Chlorella vulgaris* could reduce COD by 62% (Lim et al. 2010). In our research investigation, the COD concentrations measured after each run were consistently lower than the control value, indicating effective COD removal. This can be attributed to the oxygen generated by photosynthetic organisms and microalgae, which enhances the biological degradation of organic matter in wastewater.

### Decolourization of TWW

Decolourization is an essential process in TWW treatment. The efficiency of all the treatments in decolorizing the wastewater was assessed, except for the control group that did not contain wastewater. The results showed that T1, T2, T3, T4, and T5 achieved maximum decolourization efficiencies of 98.16 (%), 97.27 (%), 98.12 (%), 99.71 (%), and 96.18 (%), respectively. Therefore, all treatments successfully achieved nearly complete decolourization. It is worth mentioning that even in T1, T2, T3 and T5, which had a low cell concentration, still exhibited excellent decolourization capabilities. Previous research has shown that higher initial dye concentrations create concentration gradients that enhance mass transfer between dye particles and microalgae, thereby accelerating the removal rate of the dye. In the current study, the concentration gradient between dye molecules and algal cells was higher in T4 and T3. This was attributed to their slower growth rate and higher dye concentration in comparison to the other treatments. As a result, these treatments displayed a higher initial rate of dye removal (Fazal et al. 2021).

Generally, decolourization of wastewater can occur through two separate processes: bioaccumulation and biosorption. Microalgae can accumulate dye through bioaccumulation, an intracellular process, as the organic carbon is oxidized (R. Al-Tohamy et al. 2022). The pigment molecules are broken down into less complex substances. Recent research has shown that *Chlorella vulgaris* can convert mono-azo dye into aniline and degrade it by 69% (Chai et al. 2021). On the other hand, microalgae can also absorb dye from the wastewater through biosorption, facilitated by the high surface area and strong binding affinity of microalgae. The dye ions diffuse onto the solid surface of the algal biopolymer, and the presence of functional groups in extracellular polymers aids in the biosorption of dye molecules (Andrade et al. 2018; Chu and Phang 2019). Previous studies have documented the removal of Remazol Black B dye by *Chlorella vulgaris* (Cardoso et al. 2011, 2012; Jayaseelan et al. 2022). In this investigation, all treatments demonstrated good decolourization efficiencies.

### Composition of fatty acid methyl esters

**Fig. 6:**
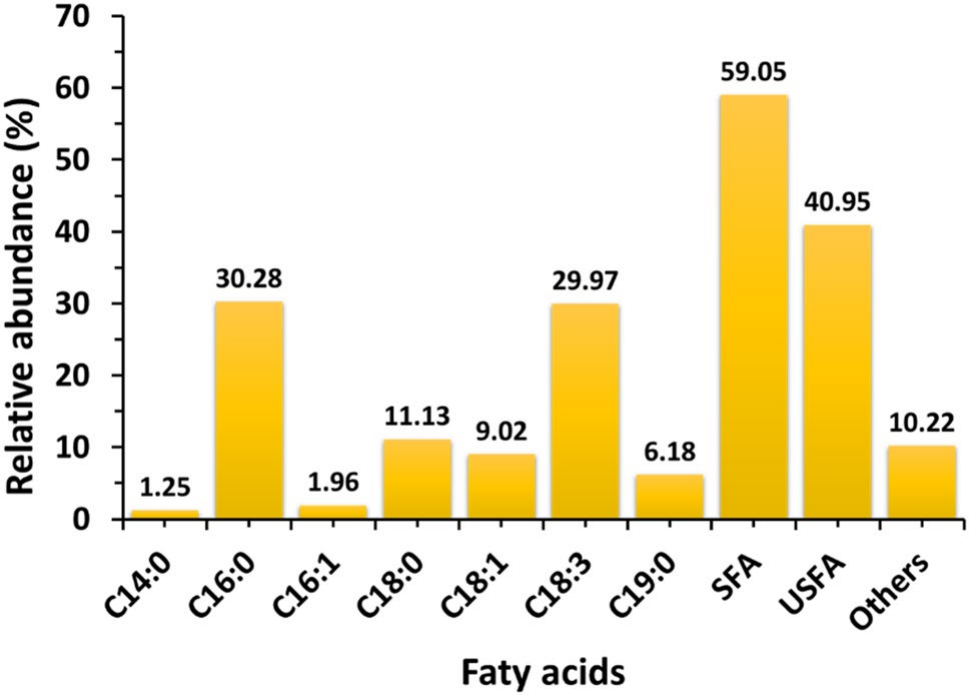
Fatty acid methyl ester composition of *Chlorella sorokiniana* ASK25 grown in recommended condition

The fatty acid methyl ester composition of the algae oil is illustrated in Fig. 6. For this investigation, the samples with the highest oil content were selected (Test T4), specifically the *Chlorella sorokiniana* isolate, ASK25 biomass yield, and lipid yield in the T4 test. The identified fatty acids in the oil included C14:0 (1.25%), C16:0 (30.28%), C16:1 (1.96%), C18:0 (11.13%), C18:1 (9.02%), C18:3 (29.97%), C19:0 (6.18%), and other fatty acids (10.22%). Saturated fatty acids (SFA) accounted for 59.05% of the composition, followed by unsaturated fatty acids (USFA) at 10.22%. The fatty acid content of the oil plays a significant role in determining the quality criteria of biodiesel. According to Liu et al. the presence of palmitic acid contributes to higher oxidative stability, cetane number, and lower NO_X_ emissions (Liu et al. 2011). On the other hand, algal oil with a high oleic acid content offers a balanced combination of fuel properties, including oxidative stability, combustion heat, viscosity, lubricity, cold filter plugging point (CFPP), and ignition quality (Santos et al. 2013). It is important to note that a high percentage of polyunsaturated fatty acids in biodiesel is undesirable due to their negative impact on fuel stability (Pandey et al. 2022). Based on these findings, the algae strains utilized in this study can be considered as potential feedstock for biodiesel production.

## Theoretical mass balance

**Fig. 7:**
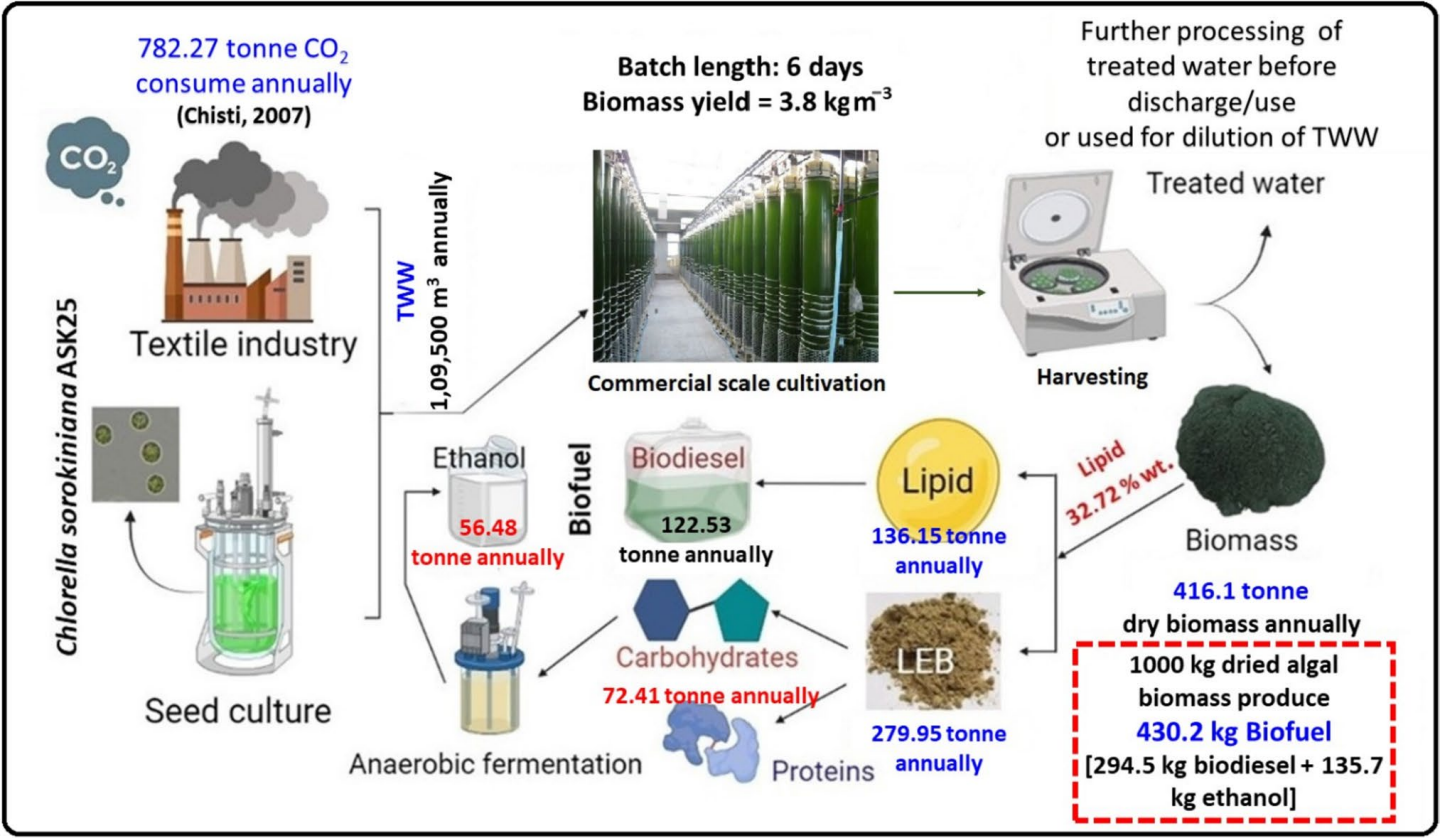
Schematic process flow for TWW biorefinery and theoretical mass balance

Microalgae require water and nutrients for their growth, making them an intriguing option for cultivating in nutrient-rich wastewater. This approach offers the dual benefit of producing a substantial amount of biomass at a low cost while simultaneously treating the wastewater. In India, the textile industry generates a significant amount of wastewater, estimated to be around 60–70 m^3^ annually (Sarayu and Sandhya 2012). With the growing demand for textiles, the production of wastewater is expected to increase accordingly. This abundance of textile wastewater provides a valuable resource for microalgae cultivation. Consequently, integrating the treatment of textile wastewater with the production of microalgal biofuels presents a promising sustainable approach (Fig. 7).

The present study demonstrated the ability of the microalga *Chlorella sorokiniana* ASK25 to efficiently assimilate nutrients, including carbon, nitrogen, and phosphorus, from TWW. It is worth noting that the textile industry in India consumes approximately 150 m^3^ of water per tonne of cotton cloth produced. Based on estimates and discussions with industry experts (anonymous), it is presumed that the daily production of cotton cloth ranges from 1 to 2 tonnes (Sarayu and Sandhya 2012; Fazal et al. 2018; Oyebamiji et al. 2019). By harnessing the potential of microalgae to utilize the nutrients (C/N/P) present in TWW, this integrated approach can contribute to both sustainable wastewater treatment and the production of valuable biofuels. The utilization of abundant textile wastewater resources, along with the capabilities of microalgae, paves the way for a more environmentally friendly and economically viable solution for the textile industry's wastewater management. Considering a daily turnover of 2 tonnes of cloth, it can be estimated that approximately 300 m^3^ of TWW is generated daily, resulting in an annual volume of 1,09,500 m^3^ (365 days). To accommodate the cultivation of *Chlorella sorokiniana* ASK25 in TWW, the construction of a commercial scale closed photobioreactor (PBR) would provide a total cultivation capacity of generated TWW (109,500 m^3^/year; 300 m^3^ d^−1^ and 6 days batch cultivation). Based on the biomass production rate of 3.80 kg m^3^, *Chlorella sorokiniana* ASK25 has the potential to produce approximately 416.1 tonnes of dry biomass annually. Moreover, considering the conversion yields of lipid to biodiesel (90%) and total sugar to fermentable sugar followed by bioethanol conversion (90% and 78%, respectively) as reported in recent studies (Neag et al. 2023; Dong et al. 2016; Mhlongo et al. 2021; Lee et al. 2015; Pancha et al. 2015), this biomass could yield approximately 122.53- and 56.48 tonnes of biodiesel and bioethanol per year, respectively. 1 tonne of dried biomass of *Chlorella sorokiniana* ASK25 could potentially yield 0.43 tonne (430.24 kg) of biofuel, comprising approximately 0.295 tonne (294.47 kg) of biodiesel and 0.135 tonne (135.74 kg) of bioethanol. Furthermore, Microalgae can sequester 1.88 kg of carbon dioxide to produce 1 kg of biomass., it is estimated that approximately 782.27 tonne of CO_2_ could be consumed annually through this process (Kumar et al. 2014; Chisti 2007). To assess the economic viability and carbon and energy balance of this integrated technique, a life cycle analysis of biomass and biofuel production from *Chlorella sorokiniana* ASK25 in TWW is necessary. The findings from this laboratory-scale investigation can inform the development of a pilot-scale inquiry.

By adopting such an integrated approach, the cost of TWW treatment could be reduced while simultaneously facilitating the production of microalgal biomass with high lipid and carbohydrate contents, which can be further utilized for biofuel production (Maurya et al. 2016; Alishah Aratboni et al. 2019). These value-added applications contribute to the overall cost reduction in microalgae-based biodiesel production. However, research opportunities lie in the development of sustainable microalgae bio-refineries, including scaling up the cultivation process, designing high-efficiency bioreactors, and developing downstream processing for value-added products such as carotenoids, cosmetics, proteins, pigments, lutein chemicals, and bioethanol, among others.

## Future prospects of this study

The utilization of microalgae in treating TWW holds great promise for advancing sustainable wastewater management practices and the production of valuable bioresources. Based on the current findings and outcomes, there are several key areas to focus on to further enhance the application of microalgae in TWW treatment. Firstly, optimizing the cultivation conditions of microalgae, specifically *Chlorella sorokiniana* ASK25, can significantly improve biomass production and lipid content. By exploring different parameters such as light intensity, temperature, pH, and nutrient concentrations, tailored cultivation strategies can be developed to maximize the efficiency of microalgae growth (Ray et al. 2021). Efforts should also be directed towards improving the nutrient removal efficiency of microalgae in TWW treatment. This involves investigating strategies to enhance the assimilation of carbon, nitrogen, and phosphorus by microalgae, as well as understanding the impact of trace elements on nutrient uptake. By optimizing nutrient utilization, the effectiveness of microalgae-based wastewater treatment systems can be further enhanced (Hussain et al. 2020). Genetic modification techniques offer another avenue for future research. By genetically engineering microalgae strains such as *Chlorella sorokiniana* ASK25, it is possible to enhance lipid productivity and stress tolerance. Manipulating key genes involved in lipid biosynthesis or stress response pathways can lead to the development of improved strains with higher lipid content and overall performance (Park et al. 2019).

Integration of microalgae-based wastewater treatment systems with other advanced treatment technologies can also be explored. Combining microalgae cultivation with anaerobic digestion or other processes can enhance the overall efficiency of wastewater treatment and facilitate the recovery of valuable resources. Investigating the synergistic effects of different treatment methods can lead to more sustainable and integrated wastewater treatment approaches (Magalhães et al. 2021). Additionally, conducting a comprehensive life cycle assessment (LCA) of microalgae-based wastewater treatment and biodiesel production is crucial for evaluating the environmental and economic sustainability of the process. LCA provides a holistic perspective on the entire life cycle, helping to identify areas for improvement and guiding decision-making in terms of process optimization and scaling up (Rajak et al. 2023). Scaling up microalgae-based wastewater treatment systems from lab-scale to pilot-scale and eventually to industrial-scale is a vital step towards practical implementation. Establishing demonstration projects can provide valuable insights into the feasibility, efficiency, and economic viability of large-scale microalgae cultivation and wastewater treatment. These projects serve as real-world tests to validate the effectiveness and reliability of microalgae-based systems (Pendyala et al. 2020). In an interesting study, Alprol et al. (2021) investigated the efficiency of *Arthrospira* complete dry biomass (ACDB) and LFB (lipid free biomass) in bioremediation of dye from TWW and also examined the potential of ACDB and LFB loaded by IV2R as a feed for Rotifer, *Brachionus plicatilis*. The adsorption process increased with increasing ACDB and LFB dose, contact time (120 min.), initial IV2R concentration (10 mg L^−1^), and acidity pH (2 and 6, respectively). The ACDB and LFB sorbents have good elimination ability of a dye solution by 75.7% and 61.11%, respectively. In addition, based on the bioassay study, the ACDB and LFB loaded by IV2R up to 0.02 per litre may be used as feed for the marine rotifer *B. plicatilis* (Alprol et al. 2021). By addressing these prospects, researchers and practitioners can further advance the application of microalgae in TWW treatment, contributing to sustainable wastewater management practices and the development of a circular economy. These efforts have the potential to not only mitigate pollution but also create valuable bioresources from wastewater, promoting a more sustainable and environmentally friendly approach to industrial wastewater treatment.

## Conclusion

In this study we show that TWW supplemented with nutrients (BG-11 medium) can be valorised by using them as the biofuel feedstock for the cultivation of unicellular green microalgae *Chlorella sorokiniana* ASK25. The immediate benefit of that was the production of the microalgal biomass, TWW treatment and decolourization of TWW. *Chlorella sorokiniana* ASK25 efficiently utilized and removed nutrients from untreated TWW without the need for extensive pre-treatments. After six days of batch cultivation, a notable decline in pollutant (organic and inorganic load) was observed, coinciding with the high level of biomass production. The generated biomass exhibited promising prospects to produce biodiesel and bioethanol, with lipid content ranging from 21.93 (%, wt.) to 32.72 (%, wt.) and carbohydrate content range from 18.70 (%, wt.) to 35.05 (%, wt.). Colour and heavy metal removal is a type of treatment that is not naturally occurring in typical wastewater treatment facilities. Therefore, combining wastewater treatment with algae cultivation has the potential to produce microalgae biomass while further improving the quality of treated wastewater. It has been demonstrated that textile effluent can generate microalgae biomass that can be utilised to generate biofuels while reducing the ecological damage posed by nutrient-rich wastewater.

## Data Availability

The datasets used and/or analysed during the current study are available from the corresponding author on reasonable request.
