# Correction to: Axenic green microalgae for the treatment of textile effluent and the production of biofuel: a promising sustainable approach

**DOI:** 10.1007/s11274-024-04024-9

**Published:** 2024-05-23

**Authors:** Ashutosh Pandey, Gaurav Kant, Ashwani Chaudhary, Kassian T. T. Amesho, Karen Reddy, Faizal Bux

**Affiliations:** 1 Institute for Water and Wastewater Technology, Durban University of Technology, 19 Steve Biko Road, Durban, 4000 South Africa; 2 Bioenergy Research Laboratory, Faculty of Life Science and Technology, Department of Biotechnology, AKS University Satna, Satna, MP 485001 India; 3 Department of Biotechnology, Motilal Nehru National Institute of Technology Allahabad, Prayagraj, UP 211004 India; 4 Department of Biotechnology, IMS Engineering College (Affiliated to Dr APJ Abdul Kalam Technical University, Lucknow), Ghaziabad, UP 201015 India; 5 Institute of Environmental Engineering, National Sun Yat-Sen University, Kaohsiung, 804 Taiwan; 6 Centre for Emerging Contaminants Research, National Sun Yat-Sen University, Kaohsiung, 804 Taiwan; 7 Centre for Environmental Studies, The International University of Management, Main Campus, Dorado Park Ext 1, Windhoek, 10001 Namibia; 8 Amity Institute of Biotechnology, Amity University, Noida Campus, Sec-125, Noida, UP 201313 India

**Correction to: World Journal of Microbiology and Biotechnology**

10.1007/s11274-023-03863-2

In the **Results and discussions** paragraph titled “**Composition of fatty acid methyl esters”** and **Fig. 6**: The relative abundance of saturated fatty acids (SFA) and others are needed to be change as

The identified fatty acids in the oil included C14:0 (1.25%), C16:0 (30.28%), C16:1 (1.96%), C18:0 (11.13%), C18:1 (9.02%), C18:3 (29.97%), C19:0 (6.18%), and other fatty acids (10.21%). Saturated fatty acids (SFA) accounted for 48.84% of the composition, followed by unsaturated fatty acids (USFA) at 40.95%.

**Fig. 1:**
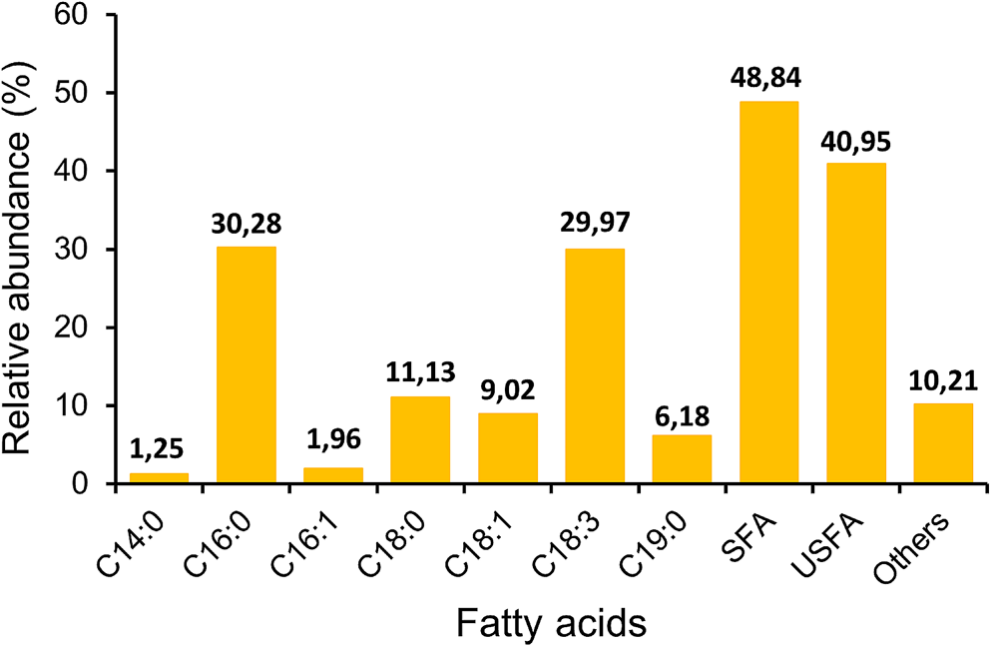
Fatty acid methyl ester composition of *Chlorella sorokiniana* ASK25 grown in recommended condition

